# Large-Scale Functional Purification of Recombinant HIV-1 Capsid

**DOI:** 10.1371/journal.pone.0058035

**Published:** 2013-03-05

**Authors:** Magdeleine Hung, Anita Niedziela-Majka, Debi Jin, Melanie Wong, Stephanie Leavitt, Katherine M. Brendza, Xiaohong Liu, Roman Sakowicz

**Affiliations:** Gilead Sciences Inc., Foster City, California, United States of America; University of California San Francisco, United States of America

## Abstract

During human immunodeficiency virus type-1 (HIV-1) virion maturation, capsid proteins undergo a major rearrangement to form a conical core that protects the viral nucleoprotein complexes. Mutations in the capsid sequence that alter the stability of the capsid core are deleterious to viral infectivity and replication. Recently, capsid assembly has become an attractive target for the development of a new generation of anti-retroviral agents. Drug screening efforts and subsequent structural and mechanistic studies require gram quantities of active, homogeneous and pure protein. Conventional means of laboratory purification of *Escherichia coli* expressed recombinant capsid protein rely on column chromatography steps that are not amenable to large-scale production. Here we present a function-based purification of wild-type and quadruple mutant capsid proteins, which relies on the inherent propensity of capsid protein to polymerize and depolymerize. This method does not require the packing of sizable chromatography columns and can generate double-digit gram quantities of functionally and biochemically well-behaved proteins with greater than 98% purity. We have used the purified capsid protein to characterize two known assembly inhibitors in our in-house developed polymerization assay and to measure their binding affinities. Our capsid purification procedure provides a robust method for purifying large quantities of a key protein in the HIV-1 life cycle, facilitating identification of the next generation anti-HIV agents.

## Introduction

Human immunodeficiency virus (HIV) infections require a lifelong therapy. Despite the availability of highly effective anti-HIV drugs, the development of drug-resistant HIV-1 variants remains a major threat to the patient population. To overcome the emergence of drug resistance during current therapies, there is a constant need for the discovery of new classes of antiretroviralsto augment existing HIV treatment regimen.

HIV capsid protein (CA) represents such a target, with biologically validated importance in HIV life cycle but requiring a clinical proof of concept. During HIV virion maturation, CA is released from the Gag polyprotein by the viral protease [1]. Despite the presence of other structural and non-structural proteins in the maturing virion, ∼1,500 CA monomers assemble into a lattice of ∼ 250 CA hexamers interspersed with ∼12 pentamers that together form a distinct fullerene cone encapsulating the viral RNA. Upon entry of HIV particles into host cells, the CA core disassembles in a coordinated fashion to allow reverse transcription and subsequent integration of the reverse-transcribed viral genome into the host DNA. Stability of the CA core and the corresponding rate of core disassembly are essential for successful viral infection [2], [3]. A number of deleterious surface mutations in CA protein were reported, that alter the infectivity, replication and assembly of virions *in vivo* [4]. Disruption of proper CA core assembly during particle maturation and/or destabilization of the incoming CA core [5], which causes premature disassembly, may enable new antiviral strategies that target an essential part in the life cycle of the HIV virus. Currently, a number of small molecules with the potential to interfere with CA assembly pathway are under development [6]. Such efforts are critically dependent on the availability of large quantities of homogeneous and active CA protein.

A typical *in vitro* CA assembly assay uses large quantities of recombinant CA protein at concentrations ranging from 60–200 µM. CA polymerization is monitored spectrophotometrically by measuring the increase in absorbance at λ_350 nm_ due to the light scattering caused by polymerized tubular structures [7]–[9]. Due to the high concentration of protein required in the assay, gram quantities of CA are needed to support high-throughput screening efforts to identify inhibitors of the polymerization process. Large amounts of CA are also essential for structural studies to supplement rational drug discovery efforts. The existing published procedures describing CA purification methods are not conducive for such large scale purification efforts. One earlier report on recombinant CA purification by Gross et al (1997) relied on multiple rounds of ammonium sulfate precipitation to separate the CA proteins from *E. coli* contaminant proteins. Later protocols adopted differential ammonium sulfate fractionation as the first step coupled with either anion exchange chromatography using Tris buffer at pH 8.1 [10] or cation exchange chromatography in KMOPS buffer at pH 6.9 [11]. As there is a limit to the dynamic binding capacity of any chromatographic medium, a sizable volume of chromatography matrix would be required to capture double-digit gram quantities of CA protein. In addition, liters of buffer would be required for the column equilibration, loading and elution. Thus, a chromatography-based approach is not amenable to gram-scale protein purification in a traditional laboratory setting. Moreover, CA protein that is purified with differential ammonium sulfate fractionation or traditional chromatography steps is refined by exploiting its biochemical properties rather than its functional competency.

Here, we describe a novel CA purification method exploiting its innate ability to polymerize and depolymerize *in vitro*. This new protocol enables rapid purification of double-digit gram quantities of pure and functional HIV-1 CA protein without the need to separate the target protein on any chromatographic medium. The final product is highly enriched in polymerization-competent capsid protein, ensuring high functional activity. We have successfully used this protein to study wild-type capsid (WT CA) polymerization, to form covalent CA hexamers [12] and to monitor the effects of known CA inhibitors.

## Materials and Methods

### Construction of Wild-Type and Mutant CA Expression Plasmids

A fragment encoding the 231 residues of wild-type HIV-1 CA protein was PCR amplified from the infectious HIV-1 cDNA plasmid, pLAI.2, obtained from Dr. J. M. Becket (Centralized Facility for AIDS Reagents, contract QLK2-CT-1999-00609). The 5′ PCR primer contained an *NdeI* restriction site and a codon for N-terminal methionine. The 3′ PCR primer contained a stop codon followed by a *XhoI* restriction site. The amplified fragment was digested with *NdeI* and *XhoI*, gel-purified using the QIAquick Gel Extraction Kit (Catalog #28704, Qiagen, Valencia, CA) and cloned into the *NdeI* and *XhoI* sites of pET30a vector (Catalog #70781, EMD BioSciences, La Jolla, CA). The plasmid expressing a quadruple mutant (A14C/E45C/W184A/M185A) CA (CA 4Mu) was based on a capsid sequence from HIV-1_NL4–3_ strain and according to the amino acid sequence of protein construct in PDB:3H47 entry [12]. This plasmid was codon-optimized for *E. coli* expression and cloned into pJexpress 411 vector harboring a T7 promoter (DNA2.0, Menlo Park, CA).

### Expression of Wild-Type and Mutant CA

Plasmids carrying wild-type (WT) or mutant (CA 4Mu) capsids were transformed into One Shot® Chemically Competent BL21 DE3 cells (Catalog #C6000-03, Invitrogen, Carlsbad, CA). 18 liters of 2× YT medium containing 50 µg/mL kanamycin were inoculated with 200 mL of an overnight starter culture and incubated at 37°C in a BioStatC Fermentor (Sartorius AG, Gottingen, Germany) until OD_600_ reached 1.0. Protein expression was induced by the addition of IPTG to a final concentration of 0.5 mM at 28°C. The induced culture was harvested 4 hours post-induction.

### Purification of Wild-Type and Mutant CA

The purification protocols for wild-type (WT) and mutant (CA 4Mu) capsids were similar, with only minor differences in buffer composition. Cell pellets containing WT CA protein were resuspended in lysis buffer (50 mM Tris-HCl pH 8.0, 5 mM β-mercaptoethanol) supplemented with 1 mM PMSF at a ratio of 3 liter/kg of cell paste. The cells were lysed by three passages through an M-110L Microfluidizer Processor (Microfluidics, Newton, MA) at 18,000 psi and then centrifuged at 12,000×g for one hour in a Beckman JLA8.100 rotor. Soluble CA protein in the clarified lysate was concentrated by the addition of ammonium sulfate to 25% saturation. The precipitate was collected by centrifugation at 12,000×g for one hour and dissolved in Resolubilization Buffer (50 mM Tris pH 8.0) at 50% of the original volume of lysis buffer using a 100 ml KONTES Dounce homogenizer. An equal volume of 5 M NaCl was then added to the solubilized pellet and the solution was left stirring for 2 hours at 4°C. The resulting precipitate of polymerized CA was collected by centrifugation at 12,000×g for one hour and resuspended in Resolubilization Buffer to allow for dissolution. An additional round of polymerization using an equal volume of 5 M NaCl was performed. After the second round of polymerization/depolymerization, WT CA protein was resuspended in 50 mM sodium phosphate pH 7.5 buffer and dialyzed against the same buffer overnight. The dialyzed CA sample was further purified from trace amounts of *E. coli* contaminating proteins by subtractive chromatography on a 5 mL Q-HP HiTrap column (Catalog #17-1154-01, GE Healthcare, Piscataway, NJ). Pure CA protein in the flow-through fraction was pooled and flash-frozen in liquid nitrogen.

Purification of CA 4Mu monomer followed the same scheme as WT CA, except that 200 mM β-mercaptoethanol was included in all buffers and ammonium sulfate fractionation was performed at 30% saturation. CA 4Mu protein harvested after a second round of polymerization was solubilized in 50 mM Tris pH 7.5 and 40 mM β-mercaptoethanol buffer prior to dialysis against the same buffer. The protein was further purified by a passage through a 5 mL anion exchange chromatography column.

### Preparation of Cross-linked CA 4Mu Hexamer

Soluble cross-linked CA 4Mu hexamers were prepared as previously described [12]. After the serial dialysis scheme, cross-linked hexamers were separated from other oligomeric species by size exclusion chromatography on a Shodex KW2003 column (Showa Denko America, New York, NY) equilibrated in 50 mM Tris-HCl pH 8.0 buffer.

### Capsid Assembly Inhibitors

CAI peptide **(**amino acid sequence: ITFEDLLDYYGP) and CAI-4 peptide (amino acid sequence: ITFEDLLDYYK) were obtained from AnaSpec Inc., Fremont, CA. BM2 [13] was contract-synthesized by Shanghai Medicilon Inc., China.

### Mass Spectrometry

Mass Spectrometry of WT CA and CA 4Mu protein samples was performed on an Agilent 6210 Time of Flight Mass Spectrometer with an Agilent 1200 Rapid Resolution HPLC. The samples were run on an Agilent Zorbax 300 Extend C18 Rapid Resolution column at 70°C, using reverse phase chromatography with a gradient from 20% to 90% acetonitrile containing 0.1% formic acid. Data were processed via Agilent Masshunter B.04 Qualitative Analysis, with the Bioconfirm upgrade, allowing for protein deconvolution. CA hexamer samples were analyzed using the same method except data collection was extended to 20,000 m/z.

### Transmission Electron Microscopy

Polymerized CA samples for transmission electron microscopy were prepared at a final concentration of 40 µM WT CA monomer as described in Capsid Polymerization Assay section of Materials and Methods. Soluble cross-linked CA 4Mu hexamers were examined at 0.5 mg/mL (19.5 µM CA monomer) in 20 mM Tris pH 8.0, 10% glycerol. Transmission electron microscopy was performed on negative stained samples. Samples were deposited onto parlodion filmed carbon-coated grids and embedded in 2% potassium phosphotungstate pH 6.5 using the drop method. Negatively stained specimens were examined at 800 kV in a JEOL JEM-1230 electron microscope (JEOL USA Inc., Peabody, MA) and photographed with a Gatan Ultrascan USC1000 digital camera (Gatan Inc., Warrendale, PA).

### Analytical Ultracentrifugation

Proteins were first dialyzed against two changes of 2 liters of 50 mM sodium phosphate pH 7.5 at 4°C over a period of 24 hours. Dialyzed proteins were filtered through 0.22 µm centrifugal filter devices (Ultrafree-MC, Millipore Corp., Bedford, MA) by centrifugation at 6,000 rpm for 5 minutes at 4°C using an Eppendorf 5414C centrifuge (Eppendorf, Hamburg, Germany). Analytical sedimentation velocity experiments were performed in a ProteomeLab XL-A analytical ultracentrifuge. Samples of a reference buffer or various concentrations of CA protein were loaded into a dual sector charcoal-filled epon centerpiece. The samples were centrifuged at 45,000 rpm in an An50Ti rotor (Beckman Coulter, Fullerton, CA) at 25°C and absorbance data were collected by scanning the sample cells at wavelength of 280 nm. Continuous sedimentation coefficient distribution, c(s), was calculated using SEDFIT program [14]–[15] as described previously [16]. Calculated apparent sedimentation coefficient values, *s,* were converted to the standard conditions of water at 20°C (s_20,w_) using SEDFIT. Buffer density and viscosity were calculated from buffer composition with tabulated values in Sedenterp [17]. Partial specific volume of protein was calculated from amino acid composition as implemented in Sedenterp.

### Capsid Polymerization Assay

Polymerization reactions for WT CA and CA 4Mu were performed as previously described [18]. Briefly, 50 µL of CA proteins at 2× final concentration (0 µM to 80 µM for WT CA and 0 µM to 250 µM for CA 4Mu) were prepared in a buffer containing 50 mM sodium phosphate pH 7.5, 0.005% (v/v) Antifoam 204 and loaded into a 96-well plate. After incubation at 25°C for 5 minutes, polymerization was initiated by the addition of 50 µL of buffer containing 50 mM sodium phosphate pH 7.5, 4 M NaCl, 0.005% (v/v) Antifoam 204. The plate was covered with a plate sealer to reduce evaporation. Extent of polymerization was monitored by measuring the increase in sample absorbance at 350 nm over the course of 12 hours at 20-second intervals at 25°C in an M5 plate reader (Molecular Devices, Sunnyvale, CA).

### Inhibition of Capsid Polymerization by Small Molecule Inhibitors

CAI-4 peptide and BM2 were initially serially diluted in 100% DMSO and intermediate compound dilutions were obtained by mixing 5 µL of DMSO dilutions with 45 µL of 50 mM sodium phosphate pH 7.5, 0.005% Antifoam 204. WT CA was diluted to 100 µM concentration in buffer containing 50 mM sodium phosphate pH 7.5, 0.005% Antifoam. 40 µL of diluted CA was mixed with 10 µL of intermediate compound dilutions (2-fold serial dilution from 20 µM final for CAI-4 peptide or 50 µM final for BM2 compound) in 50 mM sodium phosphate pH 7.5, 0.005% Antifoam 204, and the CA/inhibitor mixture was incubated at 25°C for 10 min to allow for thermal equilibration and binding of compound to the protein. CA polymerization was initiated by the addition of 50 µL 50 mM sodium phosphate pH 7.5, 4 M NaCl, 0.005% Antifoam to the pre-incubated CA/inhibitor mixture and the extent of polymerization was monitored as described above. The final reaction contained 40 µM CA and various concentrations of inhibitors in 50 mM sodium phosphate pH 7.5, 2 M NaCl, 0.005% Antifoam 204, 1% DMSO. Initial rates of polymerization reaction were calculated from the slopes of the straight line fitted to the linear portion of the kinetic curves of CA polymerization. % Activity in the presence of inhibitor was calculated as % of initial rate in the presence of inhibitor in comparison to the rate of uninhibited polymerization reaction. Competitor dose response curves *(%Activity* as a function of inhibitor concentration) were fitted to equation (1):
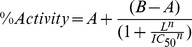
(1)where *IC_50_* is the 50% inhibitory concentration, *L* is the inhibitor concentration, *n* is the Hill coefficient, *A* and *B* are parameters related to the value of *%Activity* for fully inhibited and non-inhibited polymerization activity, respectively.

### Surface Plasmon Resonance Measurements

Surface Plasmon Resonance (SPR) measurements were performed on Biacore T100 (GE Healthcare, Piscataway, NJ) using CM5 research-grade sensor chips. Purified WT CA and assembled CA 4Mu hexamer were minimally biotinylated at a ratio of 1 biotin molecule per molecule of either monomeric or hexameric capsid using EZ-link Sulfo-NHS-LC-LC-biotin (cat#21338, ThermoScientific, Barrington, IL). All binding experiments were performed in buffer containing 50 mM sodium phosphate pH 7.5, 150 mM NaCl, 0.01% P20 (v/v). CM5 sensor chips were pre-conditioned before neutravidin was immobilized at a range between 7000 to 9000 RU on all flow-cells via standard amine-coupling chemistry (cat# BR-1000-50, GE Healthcare, Piscataway, NJ). Minimally biotinylated proteins were diluted in 1× HEPES buffered saline (pH 7.4) and injected over separate flow-cells for varying periods of time until capture intensities reached between 1000 to 2000 RU. Remaining free biotin-binding sites on the neutravidin surface were blocked with two five-minute injections of 0.5 mM EZ-Link-Amine-PEG2-Biotin at 30 µL/min. Kinetic measurements for capsid binding were performed by injecting buffer blank, BM2 and CAI at various concentrations over the captured CA proteins in the flow-cells at a rate of 100 µL/min for 30-second association followed by 60-second dissociation time. At the end of each cycle, the injection needle and tubing were cleaned with a 50% DMSO wash. A 5-point concentration series of DMSO (ranging from 0.5% to 1.5%) was injected at the beginning and the end of the experiment to generate a calibration curve to correct for excluded volume effects. Sensorgrams were double-referenced, corrected for solvent effects and then fitted using Scrubber 2.0 software (BioLogic Software, Campbell, Austria). Steady-state responses were fitted to a one-site binding model to obtain values for maximal response (*Rmax*) and equilibrium dissociation constant (*K_D_*). When there was sufficient curvature present, data were fitted to a simple 1∶1 kinetic model to obtain *Rmax* and the association (*ka*) and dissociation (*kd*) rate constants.

## Results

### Purification of Capsid Proteins Using Functional Polymerization

**Figure 1:**
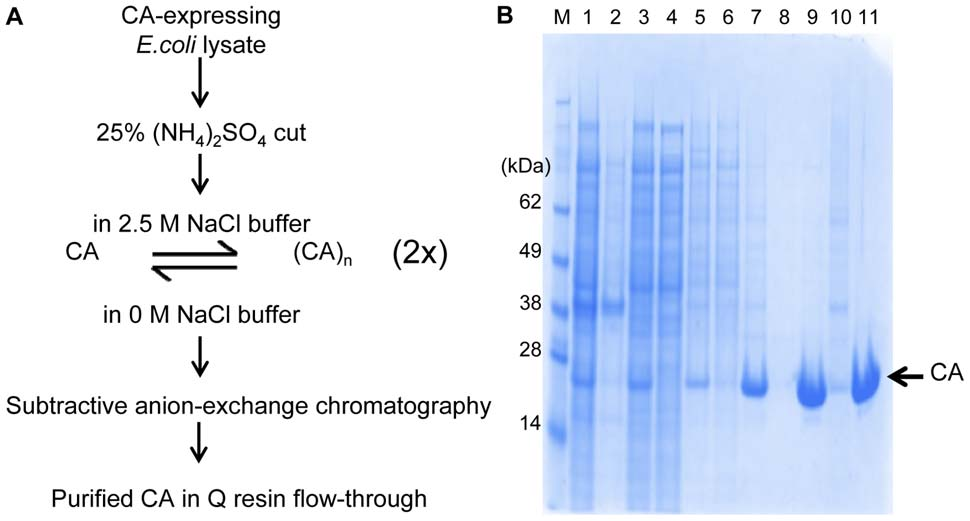
Functional purification of assembly-competent HIV-1 capsid proteins. (**A**) Flow diagram describes the functional purification scheme. Addition of NaCl to 2.5 M final concentration to the ammonium sulfate-saturated capsid solution and subsequent dissolution of the capsid-enriched pellet permits recovery of highly pure HIV-1 CA proteins after passage through the anion-exchange chromatography step. (**B**) SDS-PAGE analysis of fractions obtained during purification of wild-type capsid protein from *E. coli* lysate. The gel was stained with SimplyBlue™ Safe Stain. Lanes: M, SeeBlue Plus2 marker (the molecular weights of standards are depicted on the left-hand side of the gel); 1, total extract; 2, inclusion bodies; 3, soluble fraction; 4, supernatant after ammonium sulfate cut; 5, resolubilized 25% ammonium sulfate cut pellet; 6, unpolymerized fraction after 1^st^ addition to 2.5 M NaCl; 7, solubilized protein pellet fraction after first polymerization; 8, unpolymerized fraction after 2^nd^ addition of NaCl to 2.5 M final concentration; 9, solubilized protein pellet fraction after second polymerization; 10, insoluble pellet after dissolution of the polymerized capsid pellet from second round of polymerization; 11, Anion exchange chromatography flow-through of CA. Identity of the purified protein is confirmed by Time of Flight Mass Spectrometry (Figure S4).

Extracts from WT CA and CA 4Mu expressing *E. coli* BL21(DE3) cells induced with IPTG showed a prominent band when analyzed on SDS-PAGE gel, matching the apparent molecular weight of the expected capsid proteins (Figure S1). To purify recombinant CA protein, we initially followed the protocol established by Lanman *et al* (2002), which utilized anion exchange chromatography to fractionate the ammonium sulfate concentrated lysate. However, in order to generate double-digit gram quantities of purified CA protein using this protocol, it would require kilograms of recombinant CA expressing *E. coli* cell pellet and hundreds of milliliters of chromatographic resin. To overcome the inherent capacity issues associated with the column fractionation procedure, we developed a new purification protocol that takes advantage of the ability of HIV-1 CA protein to self-assemble under conditions of high ionic strength and high protein concentrations [19]. This functional purification method enabled us to isolate large quantities of functional CA protein without the use of chromatographic matrices for protein capture (Figure 1A). The ammonium sulfate fractionation step could effectively generate an enriched fraction of CA protein from the total lysate as previously reported (Figure 1B, Lane 5). After addition of NaCl to a final concentration of 2.5 M to the solubilized ammonium sulfate-precipitated material, CA protein readily polymerized and could be isolated from the turbid solution by a simple centrifugation step at 12,000×g. The resulting pellet contained ∼95% pure CA protein, whereas the bulk of protein contaminants remained soluble under these experimental conditions (Figure 1B, lane 6). Most importantly, the opaque pellet which contained the assembled CA protein readily depolymerized upon solubilization by resuspension in low ionic strength buffer (Figure 1B, lane 7). We subjected the first depolymerized CA pool to a second round of polymerization to further enrich for functionally active CA protein and remove any residual contaminant proteins that were still present after the first polymerization event. We noticed that the additional polymerization/depolymerization steps resulted in continued improvement in the purity of the sample (Figure 1B, lane 9). There was also a small amount of CA protein remaining in the supernatant after the polymerized CA pellet was collected, and this fraction could represent assembly-incompetent species (Figure 1B, lane 6 and 8). To improve purity of the final enriched sample, the second round of depolymerized CA material was dialyzed against 50 mM sodium phosphate buffer pH 7.5 and then passed through a 5 mL HiTrap Q-HP anionic exchanger (GE Healthcare, Piscataway, NJ) (Figure 1B, lane 11) [20]. At pH 7.5, CA protein did not bind to the anion exchange matrix and flowed through the column, whereas the contaminating bacterial proteins were efficiently captured and could be subsequently eluted from the resin using a NaCl gradient (Figure S2). Using this functional purification scheme, we were able to generate ∼10–14 grams of 98% pure CA protein from 2 kg of cell paste in 3 days without fractionation of protein over chromatographic resin. Table 1 summarizes the yield and recovery of WT CA in a typical round of purification by this procedure.

**Table 1:** Process summary of WT CA purification by polymerization.

Sample	Conc (mg/mL)	Vol (mL)	Total protein (g)	% Purity^1^	CA yield (g)
Total cell lysate	28.1	6000	168.8	15.5	26.2
ammonium sulfate fraction	7.9	4800	38.1	69.1	26.3
1st polymerized fraction	11.2	1600	17.9	94.4	16.9
2nd polymerized fraction	24.7	485	12.0	98.0	11.7
Q-sepharose flow-through	21.3	490	10.5	98.3	10.3

1 determined using HT Protein Express LabChip in Caliper LabChip GXII system.

We were able to employ the same functional purification scheme developed for WT CA to isolate the CA 4Mu monomers, which are the building blocks of the cross-linked hexamer [12] - one of the two multimeric CA structures currently available. Although the majority of the CA 4Mu protein in the ammonium sulfate saturated fraction polymerized in the presence of 2.5 M NaCl buffer, there was a significant delay in the onset of polymerization as judged by the lengthening of the time required for the solution to become visibly turbid in comparison to the wild-type protein. Therefore, each round of polymerization was allowed to continue for an extended period of time, preferably overnight, to incorporate all polymerization competent proteins into the polymer. When we attempted to solubilize the polymerized CA 4Mu in resolubilization buffer without a reducing agent, the polymerized mutant capsid did not readily dissolve. Hence the reduced ionic strength which induced disassembly of WT CA polymers was not sufficient to depolymerize the CA 4Mu polymers. Unlike the wild-type protein, the mutant CA contains engineered cysteine substitutions for residues A14 and E45 [12]. Upon assembly of mutant CA into tubular structures, these engineered cysteine residues were brought into close proximity and could form disulfide bonds. The higher stability of oligomeric structures containing cross-linked cysteine residues prevents dissolution of the mutant CA polymers in solubilization buffer in the absence of a reducing agent. However, we were able to achieve complete disassembly of the CA 4Mu polymers by solubilizing the mutant CA pellet in a buffer containing high concentration of reducing agents such as 200 mM β-mercaptoethanol [12]. Similar to the WT CA purification scheme, there was also a small fraction of CA 4Mu proteins which were unable to polymerize under the experimental conditions and remained in the supernatant after each round of polymerization. The purity of the final mutant CA sample was increased to >98% after the subtractive anion-exchange chromatography step that removed the residual bacterial contaminant proteins (Figure S3). Using a method that is based on the functional CA assembly/disassembly properties, gram quantities of CA 4Mu monomers can be prepared similarly as WT CA within short period of time and with minimal investment in chromatographic resources. In fact, we were able to generate around 2–3 times more CA 4Mu monomers than WT CA from a similar amount of cell paste. This could be due to higher expression of the mutant construct with codon usage optimized for *E. coli* cells. Identity of all purified proteins were confirmed by mass spectrometry (Figure S4).

We were able to use this functionally purified CA 4Mu monomer to form soluble hexamers following the sequential dialysis scheme described by Pornillos *et al* (2009). The assembled hexamers behaved similarly to hexamers prepared from monomer capsid proteins purified by conventional means (see below). We were also able to obtain crystals of hexameric capsid that diffracted to a similar resolution as previously reported (data not shown).

### Biophysical Characterization of Wild-type Capsid and Assembled Hexamer

**Figure 2:**
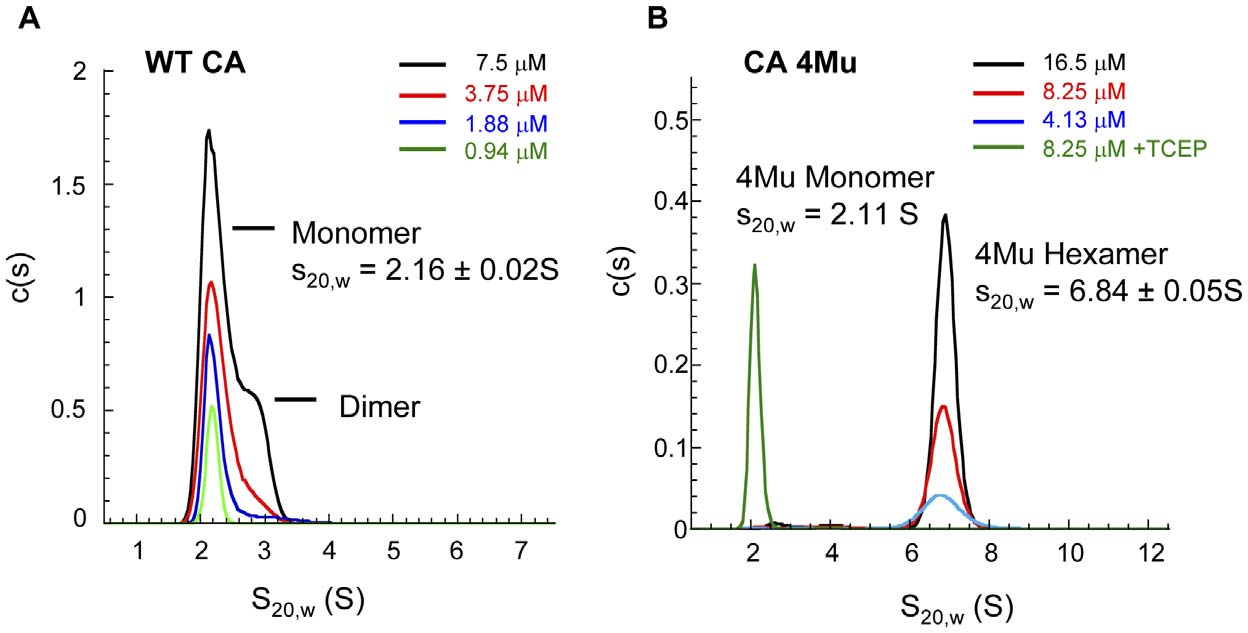
Solution oligomeric state of functionally purified WT CA and assembled CA 4Mu hexamer. (**A**) Full-length wild-type capsid protein (WT CA) and (**B**) hexameric capsid construct obtained by disulfide cross-linking of CA 4Mu monomer ([A14C,E45C,W184A,M185A]CA). Sedimentation velocity experiments were performed in 50 mM sodium phosphate buffer (pH 7.5) at 25°C and 45,000 rpm. Absorbance scans at 280 nm were collected for CA FL (**A**) at 0.94 µM, 1.88 µM, 3.75 µM, and 7.5 µM, and for CA 4Mu Hexamer (**B**) at 4.13 µM, 8.25 µM (±1 mM TCEP) and 16.5 µM (expressed as monomers of CA 4Mu). Sedimentation coefficient distribution – c(s) - was calculated using Sedfit program. WT CA contains monomers in equilibrium with dimers, whereas CA 4Mu hexamer preparation contains 94% of 4Mu monomers in hexameric form and 6% as a mixture of monomers, dimers and trimers. Treatment with 1 mM TCEP converts hexamers back to 4Mu CA monomers.

To ensure that the proteins generated by the functional purification scheme had equivalent biophysical properties as those purified by conventional means, we first characterized the oligomeric state of WT CA and cross-linked CA 4Mu hexamer in analytical sedimentation velocity experiment. Results demonstrated that WT CA existed in monomer/dimer equilibrium with species distribution dependent on protein concentration (Figure 2A), such that at high protein concentration more dimers were present while at low protein concentration, WT CA remained as a monomeric species. The protein concentration dependence on WT CA dimerization was studied previously and *K_D_* of dimer formation was determined to be 10–18 µM [21]. On the other hand, the assembled CA 4Mu protein displayed a single symmetrical peak with unchanged position regardless of protein concentration and with calculated sedimentation coefficient and molecular weight characteristic of a hexamer (Figure 2B). The hexamer preparation contained 94% of CA 4Mu proteins in hexameric form and 6% as a mixture of monomers, dimers and trimers. Reduction of the sample by addition of 1 mM TCEP converted the hexamers completely back to monomeric form. Mass spectrometry analysis also confirmed that each assembled hexamer was held together by 6 disulfide bonds (Figure S4).

### Capsid Protein is Competent in Polymerization Reaction

**Figure 3:**
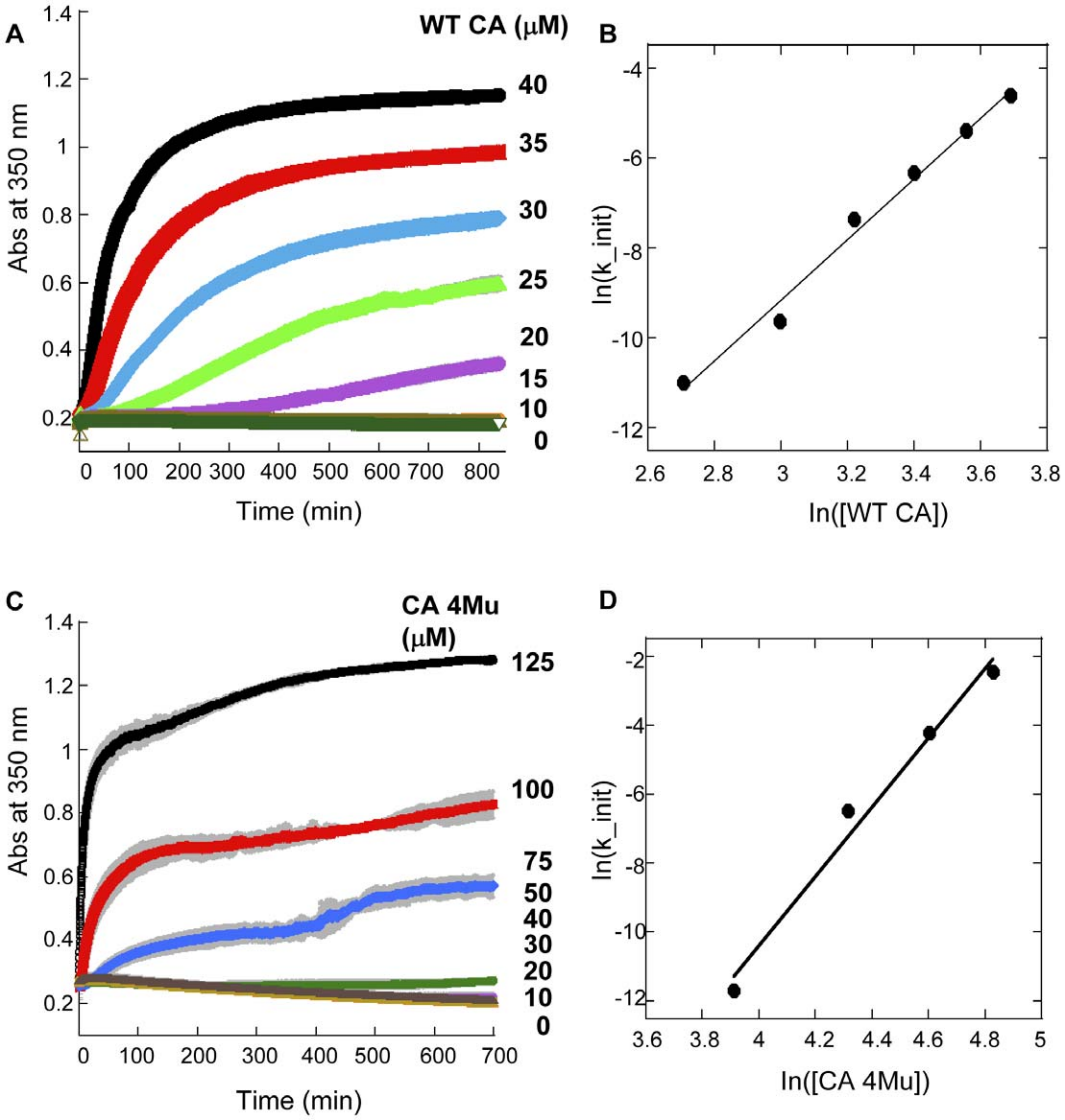
Polymerization kinetics of (A) full-length wild type capsid (WT CA) and (C) mutant capsid [A14C,E45C,W184A,M185A] monomer (CA 4Mu). Assembly reactions were induced by 2-fold dilution of capsid protein into buffer containing 50 mM sodium phosphate (pH 7.5), 4 M NaCl at 25°C. The increase in samples turbidity (absorbance at 350 nm) due to the formation of large oligomeric structures was plotted as a function of time. Final concentration of capsid protein in reaction is depicted on the right side of the kinetic traces. The data points represent an average of three determinations with standard deviation shown as grey bars. The dependence of the initial rate of capsid polymerization is plotted against the concentration of (**B**) WT CA and (**D**) CA 4Mu monomer. The initial rate was calculated from the slope of the linear phase of the polymerization curve.

Another key assessment of the quality of the CA protein was its competency to polymerize in an *in vitro* assay. To evaluate the kinetics of assembly of the functionally-purified WT CA and CA 4Mu, the proteins were allowed to polymerize under high salt condition and the change in turbidity over time (as measured by absorbance at 350 nm) was monitored. The kinetic traces of WT CA exhibited a sigmoidal shape (Figure 3A). We also noticed that when WT CA concentration was below 30 µM, the polymerization trace displayed a significant lag phase, characteristic of processes that require a nucleation step [22]–[23]. The final turbidity reading was also dependent on the starting protein concentration. Thus this functionally purified WT CA exhibited the same assembly characteristic as those purified using conventional methods [10]. When we set up the same turbidity assay for CA 4Mu, we observed a similar protein concentration-dependence on the rate and the extent of polymerization (Figure 3C). However, when CA 4Mu was tested in the same concentration range as wild-type protein (<50 µM), there was no significant change in turbidity over the course of 12 hrs. Only when the concentrations of CA 4Mu used were ≥ 75 µM, the kinetic traces showed the characteristic sigmoidal shape. When we plotted the logarithm of the initial rate of turbidity change as a function of CA concentration, we could observe a linear dose relationship for both proteins (Figure 3B and 3D). The quadruple mutant was able to achieve a similar rate of assembly as the wild-type protein at around 2.5-fold higher concentration.

**Figure 4:**
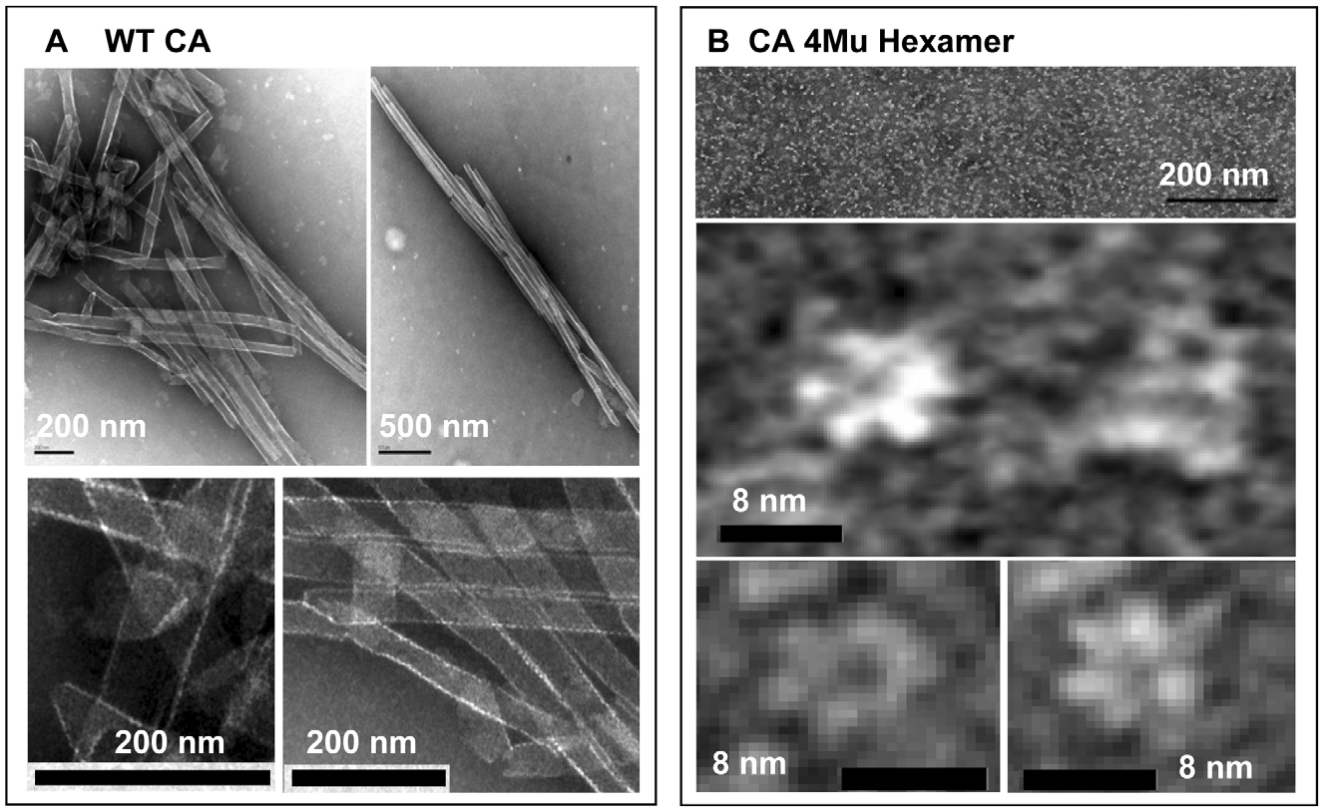
Transmission electron microscopy analysis of structures present in the solution of (A) polymerized WT CA and (B) assembled CA 4Mu Hexamer. Objects were deposited onto the grid and visualized by the negative staining. Black bar on micrographs represents scale in nm. (**A**) Tube-like and cone-like structures can be observed in solution of WT CA polymerized at 40 µM concentration in buffer containing 50 mM sodium phosphate (pH 7.5), 2 M NaCl, 0.005% Antifoam 204. (**B**) Hexameric assemblies are clearly visible in a solution containing 19.5 µM of disulfide cross-linked CA 4Mu (expressed as CA monomers concentration) in 20 mM Tris (pH 8.0), 10% glycerol buffer.

When the polymerized wild-type CA tubes were studied by transmission electron microscopy (TEM), we observed tube-like and cone-like structures (Figure 4A) which resembled the tubular structures observed for column-purified CA proteins polymerized *in vitro* [7]. The average diameter of CA tubes was 44±1 nm while the average length was 551±208 nm (average of three independent determination from analyzing more than 500 objects). On the other hand, the cross-linked CA 4Mu hexamer showed ring-like structures with an average diameter of 8±1 nm (Figure 4B) when analyzed by TEM.

### CA Protein is Sensitive to Known Assembly Inhibitors

**Figure 5:**
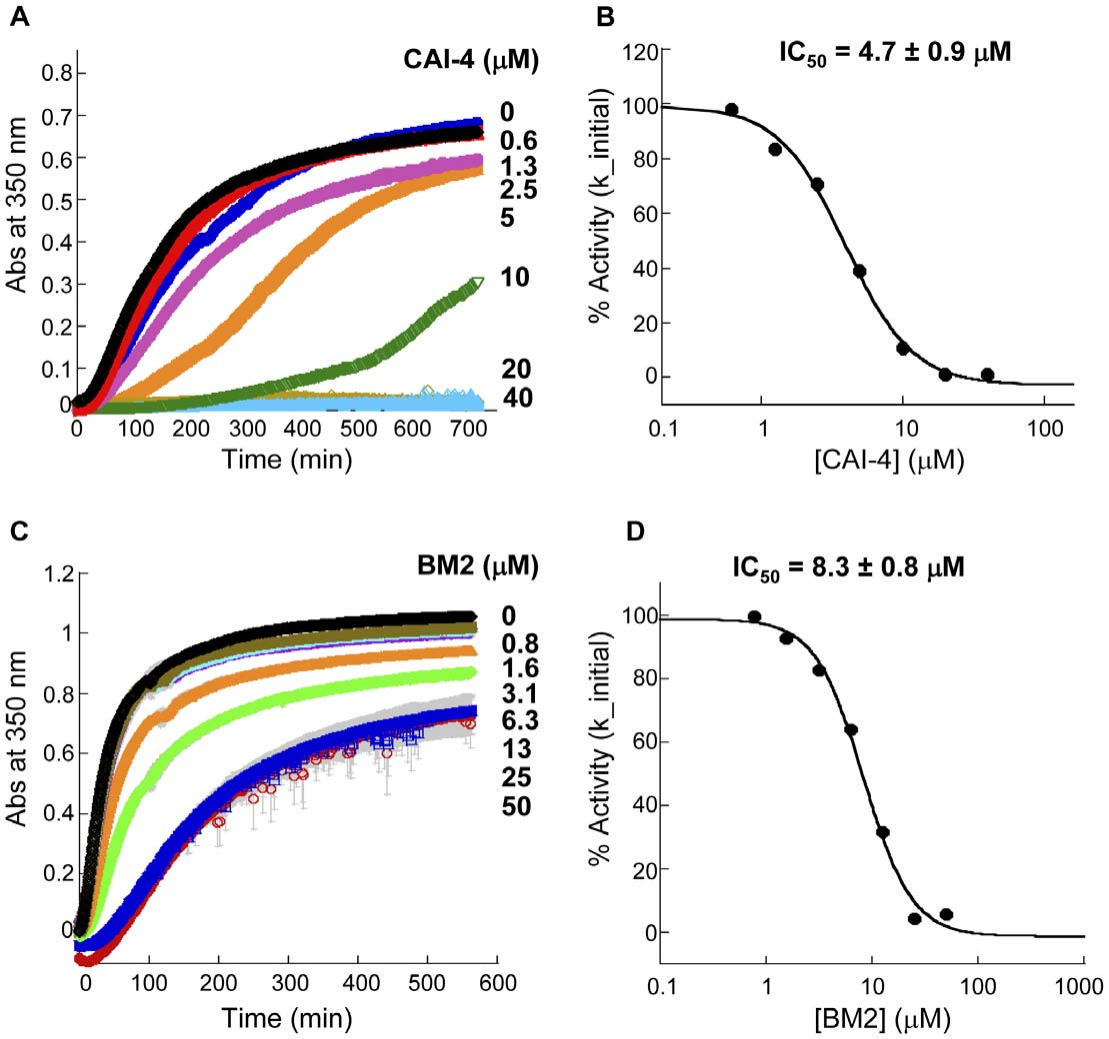
Inhibition of full-length wild-type capsid polymerization kinetics by two CA binding agents. (**A**, **B**) CAI-4 is a peptide that binds to the C-terminal domain of CA. (**C**, **D**) BM2 is a small molecule inhibitor described by Boehringer Ingelheim that binds to the CA N-terminal domain. WT CA was pre-incubated with each inhibitor. Assembly reactions were induced by 2-fold dilution of capsid protein into buffer containing 50 mM sodium phosphate pH 7.5, 4 M NaCl at 25°C. The increase of turbidity (absorbance at 350 nm) of 40 µM solution of WT CA in the presence of increasing concentrations of inhibitor (depicted on the right side of kinetic traces) was plotted as a function of time. The data points represent an average of three determinations with standard deviation shown as grey bars. The dose response curves of inhibition of polymerization kinetics and the concentration of inhibitor resulting in a half-maximal inhibition (IC_50_) for (**B**) CAI-4 and (**D**) BM2. The percent of inhibitor activity (% Activity) represents the percent of decrease of the initial rate of polymerization reaction.

To further confirm that these functionally purified proteins could be used to identify inhibitors of CA assembly, we performed the standard turbidity assay [18] to characterize the extent of inhibition of capsid polymerization by two known capsid assembly inhibitors: CAI-4 and BM2. CAI-4 is a variant of CAI peptide [24], [25], which has a lysine residue replacing the last two C-terminal amino acids, and displays similar binding affinity as full-length CAI peptide inhibitor towards wild-type CA (data not shown). BM2 is a small molecule inhibitor that was identified by Boehringer Ingelheim in an *in vitro* CA assembly assay utilizing CA-NC protein [13]. This series of inhibitors was found to bind to an induced pocket in the N-terminal domain of CA [13]. In the presence of CAI-4 and BM2, the initial rate and the extent of polymer formation in our assay decreased in a dose-dependent manner (Figures 5A and 5C), with IC_50_ values of 4.7±0.9 µM and 8.3±0.8 µM, respectively (Figures 5B and 5D).

### Characterization of Inhibitor Binding

We also employed surface plasmon resonance (SPR) technology to measure the affinity of purified proteins towards CAI and BM2, and to understand how the binding affinity (*K_D_*) correlated with their inhibitory effect on CA polymerization. WT CA monomers and assembled CA 4Mu hexamers were minimally biotinylated and captured onto the biosensor chips with immobilized layer of neutravidin. The binding of CAI and BM2 to the proteins were studied in buffer containing physiologically relevant salt concentration (150 mM of sodium chloride).

**Figure 6:**
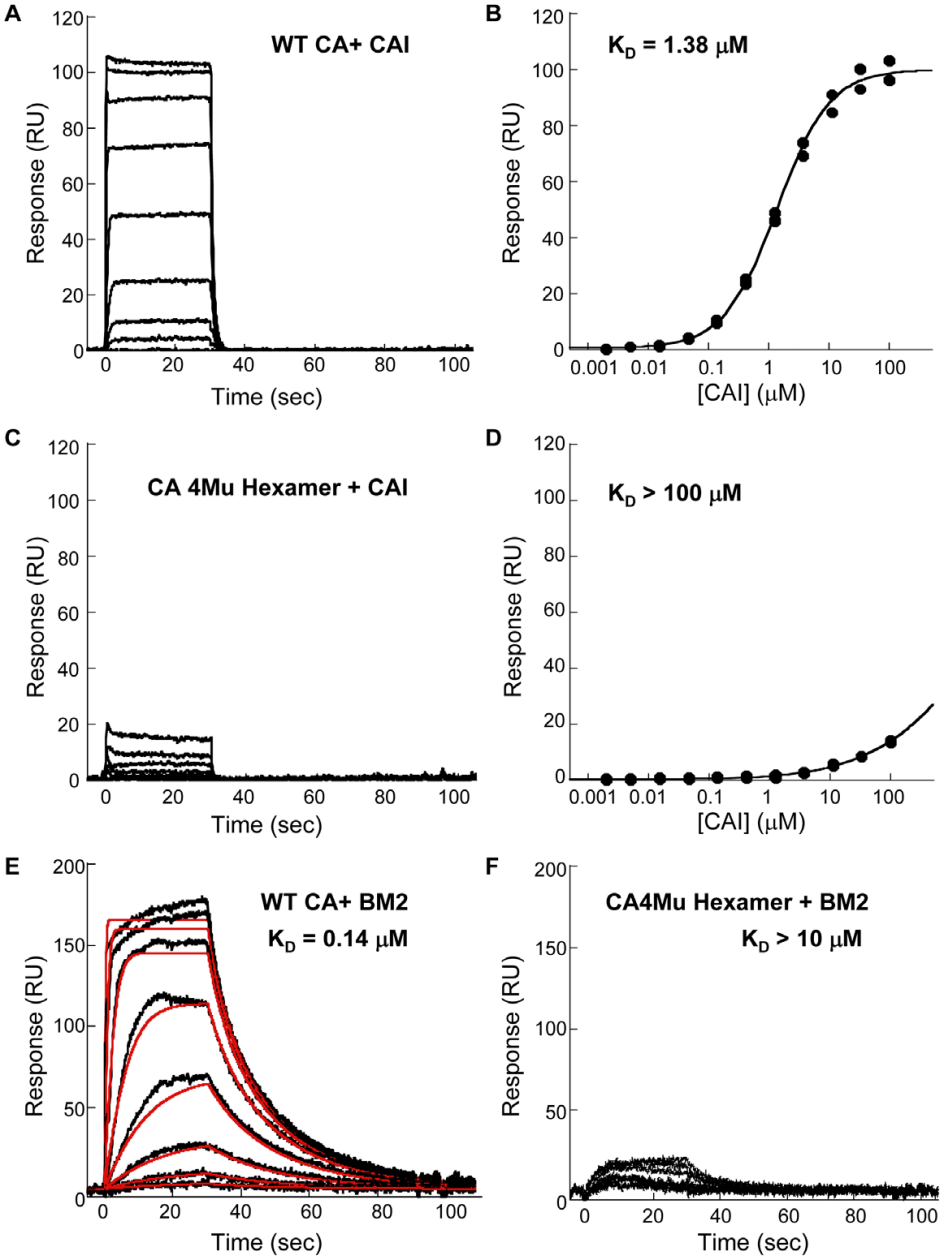
Binding of CAI peptide and BM2 to WT CA and CA 4Mu Hexamer analyzed by Surface Plasmon Resonance. (**A**) Sensorgram data of CAI binding to WT CA. CAI was injected at the highest concentration of 100 µM and ten 3-fold serial dilutions thereof. Each trace was collected in duplicate. (**B**) Fit of sensorgram data in A by fitting the equilibrium response at each concentration to a simple 1∶1 binding isotherm model. (**C**) Sensorgram data of CAI binding to hexamer. CAI concentrations used were the same as in A. (**D**) Attempt to fit sensorgram data in C to a simple 1∶1 binding isotherm model. Due to insufficient response, an accurate equilibrium dissociation constant for this interaction could not be obtained. The K_D_ for this interaction was simply cited as >100 µM. (**E**) Sensorgram data for the binding of BM2 to WT CA (black curve, sensorgram: red curve kinetic analysis using a simple 1∶1 kinetic binding model). The K_D_ for this interaction was calculated from the ratio of the k*_on_* and k*_off_* rate constants obtained from this fit. (**F**) Sensorgram data for the binding of BM2 to CA 4Mu Hexamer. The affinity of BM2 to hexamer is too weak to allow for quantification of the k*_on_* and k*_off_* rate constants.

As shown in the sensorgram in Figure 6A, CAI was found to bind to WT CA with association and dissociation rates that were too fast to be accurately determined by SPR technology, precluding kinetic analysis of the binding event. The equilibrium binding constant could be quantified from the equilibrium binding responses at different CA concentrations and fitting the data to a single site binding isotherm; this yielded an equilibrium dissociation constant of 1.38 µM (Figure 6B). On the contrary, there was only negligible binding of CAI to the assembled hexamer as shown by the lack of signal even at the highest peptide concentration (100 µM) (Figure 6C). As a result, we estimated the equilibrium dissociation constant for binding of CAI to hexamer as >100 µM (Figure 6D).

Different from the binding kinetics of CAI peptide, the small molecule CA polymerization inhibitor, BM2, was found to bind to CA monomer with a fast association rate but a visibly slower dissociation rate (Figure 6E). For BM2 binding to WT CA, there was sufficient curvature present in the data set which allowed a fit to a simple 1∶1 kinetic model. The *K_D_* derived from this kinetic fit was 0.14 µM. The tighter binding of BM2 in comparison to CAI peptide towards CA monomer was driven mainly by the slower dissociation rate. Similar to CAI, we observed negligible binding of BM2 to the assembled hexamer with estimated affinity >10 µM, which was the highest concentration of the compound tested due to limited compound solubility (Figure 6F).

## Discussion

Here we describe a method to purify HIV-1 CA protein by exploiting its ability to reversibly polymerize. This method facilitates the purification of CA protein from *E. coli* lysate without investment in bulk chromatographic resins normally used for protein fractionation. Repeated cycles of polymerization/depolymerization had been previously employed for efficient purification of other polymeric proteins like actin or tubulin from animal tissues [26]. Moreover, the incorporation of two cycles of CA polymerization and depolymerization ensured that only functional CA was purified. Unlike column fractionation, CA protein purified by this novel purification scheme can be prepared at relatively high concentrations of ∼20 mg/mL without the need of downstream concentration steps as the protein can be directly solubilized in the desired type and volume of buffer and the purity is maintained at >98% level.

We can further extend this polymerization/depolymerization approach to the purification of large quantities of the CA 4Mu ([A14C/E45C/W184A/M185A] CA). The W184A and M185A mutations in the C-domain dimerization interface were reported to significantly decrease the ability of the CA protein to dimerize and consequently to impair cylinder formation *in vitro* [4], [21], [27]. However, it was also reported that the CA 4Mu monomers were able to form tubes at much higher protein concentration but with lower efficiency [28]. As observed from our assembly kinetics experiment for CA 4Mu (Figures 4C and 4D), the weakened dimer interface affinity could be compensated by the use of higher protein concentration (>75 µM) and the initial rates of polymerization at higher CA 4Mu concentrations were comparable to the rates of WT CA polymerization. Thus we were able to use the same functional purification scheme to isolate CA 4Mu from *E. coli* lysate by maintaining a significantly high protein concentration (>10 mg/mL) throughout the process. In addition, the ability of CA 4Mu to polymerize could be enhanced by the formation of disulfide bonds between the engineered cysteine residues in the amino-terminal domain (NTD). This was further supported by the observation that we could achieve complete dissolution of the polymerized tubes formed by the quadruple mutant only in the presence of reducing agent in resolubilization buffer, for example 200 mM β-mercaptoethanol. It is worth noting that CA proteins with only C-terminal domain (CTD) dimerization interface mutations (W184A/M185A CA) were unable to polymerize under identical conditions (data not shown) and we cannot adopt the functional purification scheme in isolation of this recombinant protein.

We showed that both the wild-type and quadruple mutant CA proteins purified with this novel protocol were functionally active in the polymerization assay and the assembly process was attenuated by two well-described polymerization inhibitors: CAI-4, a variant of CAI peptide and the small molecule inhibitor BM2. Although we obtained ∼ 1 log higher IC_50_ values than previously reported [13], [24], this could be attributed to differences in assay formats employed in the determination of compound inhibition on capsid assembly, such as the type and the amount of capsid proteins.

In addition, we utilized the purified WT CA as well as the assembled CA 4Mu hexamer to measure the binding affinity to the two inhibitors using SPR methods. Although both CAI-4 peptide and BM2 inhibited CA polymerization with similar single digit micromolar potencies, there was a 10-fold difference in their respective binding affinities to the wild-type protein as shown in Figure 6B and 6E. It would be interesting to see if there is any correlation of their binding affinities to the cellular potencies. Due to the lack of cell permeability of CAI peptide, there was no direct measurement of its *in vivo* efficacy. However, there was a reasonable correlation between the dissociation constants of small molecules binding to CA_NTD_ and their respective antiviral potencies (EC_50_ values) [13]. With the availability of large amount of well-behaved protein, the SPR assay could serve as an alternative, higher throughput tool to rank potency of capsid assembly inhibitors by characterizing their binding affinities to WT CA.

Interestingly, although both inhibitors display micromolar potencies in inhibition of CA polymerization and micromolar equilibrium dissociation constants for binding to wild-type CA, they do not bind to assembled hexamer protein. Based on the structures of CA_NTD_ and CA_CTD_ domains bound to BM2 and CAI respectively, as well as modeling of the complexes in the assembled CA, their binding would sterically hinder the formation of interface contacts that would be necessary for the formation of higher-order assembled CA structures [13], [24], [25]. Thus, it would not be surprising to see a loss in the abilities of BM2 and CAI to bind to the pre-formed hexamers as the newly formed intermolecular contacts in the hexameric structure might preclude accessibility of the binding pockets to the respective inhibitors [12]. Instead, the hexameric structure would present new interfaces for the interaction with novel classes of small molecules inhibitors. In fact, we have preliminary evidence that a series of CA assembly accelerators, which is represented by PF-3450074 [29], actually displays stronger affinity to the hexamer over wild-type CA monomer (data not shown). Binding of PF-3450074 to CA_NTD_ and wild-type CA have been previously determined by isothermal titration calorimetry with comparable single-digit micromolar affinities [29]. Although there is no published data of its binding to capsid hexamer, the model of PF-3450074 in complex with capsid hexamer indicates its potential role in modulating inter-subunit interactions [29]. Hence, the differential binding affinities towards WT CA and the 4Mu hexamer demonstrated by these two classes of CA assembly modulators as measured by the SPR assay could provide an insight into the mode of action of various classes of small molecules.

In conclusion, we have developed a fast and functional approach to purify large quantities of HIV CA protein using its assembly competency. The wild-type CA proteins can subsequently be used in a high-throughput screening campaign to identify new chemical matter that may potentially become the next generation of assembly inhibitors. The quadruple mutant CA can be assembled into a hexamer and employed in the SPR binding assay to evaluate the binding affinity of inhibitors towards hexameric CA structures as part of the mode of action characterization during drug discovery effort. This new purification method will provide a significant boost to the field of HIV CA research by enabling the generation of abundant starting material for biochemical, biophysical and structural studies.

## Supporting Information

Figure S1 **Expression of WT CA and CA 4Mu in** ***E.coli*** **BL21(DE3).** *E.coli* culture samples were collected prior to induction and at the time of harvest to prepare for SDS-PAGE analysis. Total cell lysate was loaded at equivalent cell density of *E.coli* culture expressing (**A**) WT CA and (**B**) CA 4Mu. Lane M: SeeBluePlus 2 marker; Lane 1, Pre-induced sample; Lane 2, Post-induced sample.
(TIF)

Figure S2 **Final subtractive anion-exchange polishing step in capsid purification protocol.** (**A**) Chromatograhic loading and elution profile of a 5 ml Q-HP Hitrap column. The column was pre-equilibrated in 50 mM sodium phosphate buffer pH 7.5 prior to loading of 400 ml of resuspended WT CA after second round of polymerization/depolymerization and dialysis. CA protein flowed through the column and was pooled. (**B**) SDS-PAGE analysis of protein samples collected during the anion exchange step. Lane M: SeeBluePlus 2 marker; Lane 1, Q Load; Lane 2, Q flow-through; Lanes 3–6: proteins which were captured on the column and eluted later with an increasing salt gradient.
(TIF)

Figure S3 **SDS-PAGE analysis of the final pool of CA 4Mu monomer ([A14C,E45C,W184A,M185A]CA).** Lane M, SeeBluePlus2 Marker; Lane 1, Q column Load; Lane 2, Q-Flow-through.
(TIF)

Figure S4 **Mass spectrometry analysis of purified capsid proteins.** All proteins were analyzed by ESI-ToF. Molar mass determined by mass spectrometry analysis was in excellent agreement with the mass calculated based on amino acid sequence of each protein. (A) WT CA has a calculated molecular weight of 25,579.67 Da. (B) CA 4Mu monomer has a calculated molecular weight of 25,433.55 Da. (C) CA 4Mu Hexamer has a calculated molecular weight of 152,589.3 Da, confirming the formation of 6 disulfide bond.
(TIF)
