# Supplementary figures and images for: Large-Scale Functional Purification of Recombinant HIV-1 Capsid
Source: PLoS One. 2013 Mar 5;8(3):e58035. doi: 10.1371/journal.pone.0058035 (PMC3589475; doi:10.1371/journal.pone.0058035)

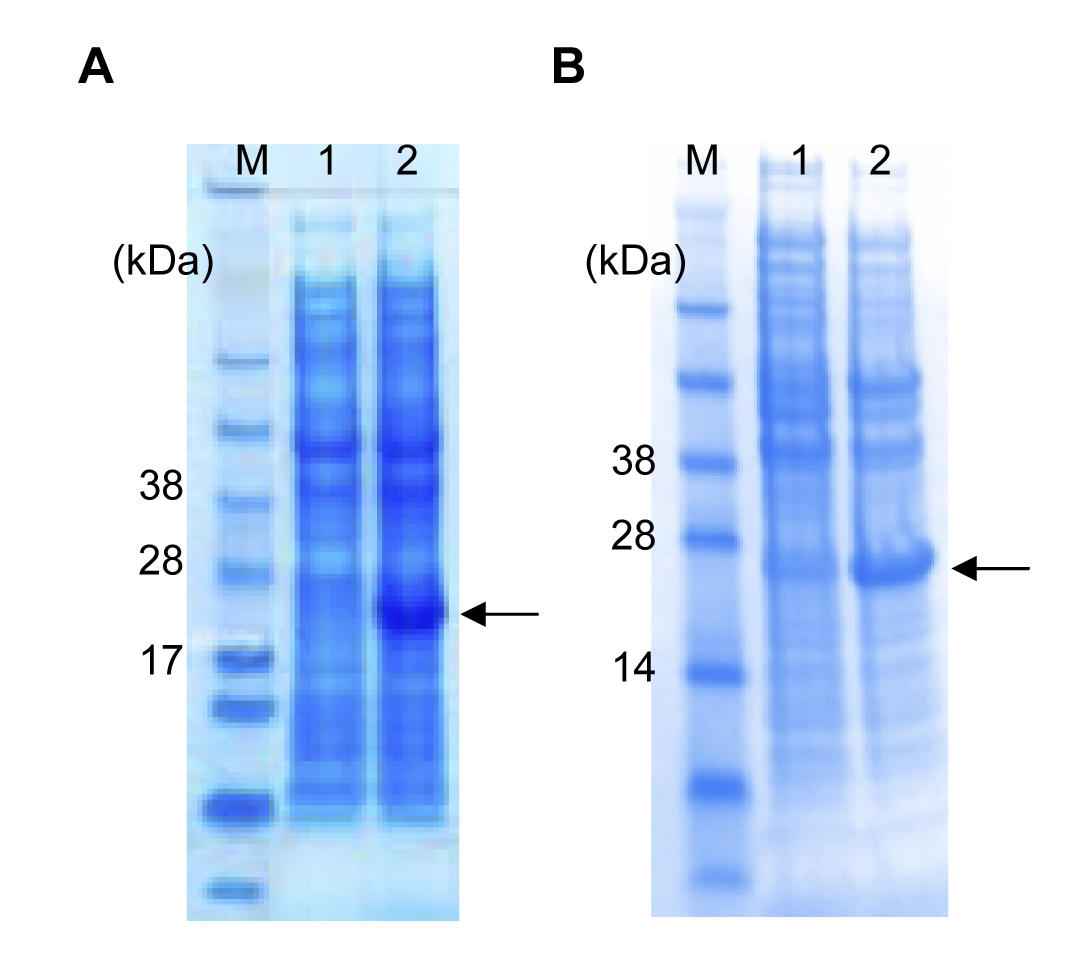

Supplement: Figure S1 — Expression of WT CA and CA 4Mu in E.coli BL21(DE3). E.coli culture samples were collected prior to induction and at the time of harvest to prepare for SDS-PAGE analysis. Total cell lysate was loaded at equivalent cell density of E.coli culture expressing (A) WT CA and (B) CA 4Mu. Lane M: SeeBluePlus 2 marker; Lane 1, Pre-induced sample; Lane 2, Post-induced sample. (TIF) [file pone.0058035.s001.tif]

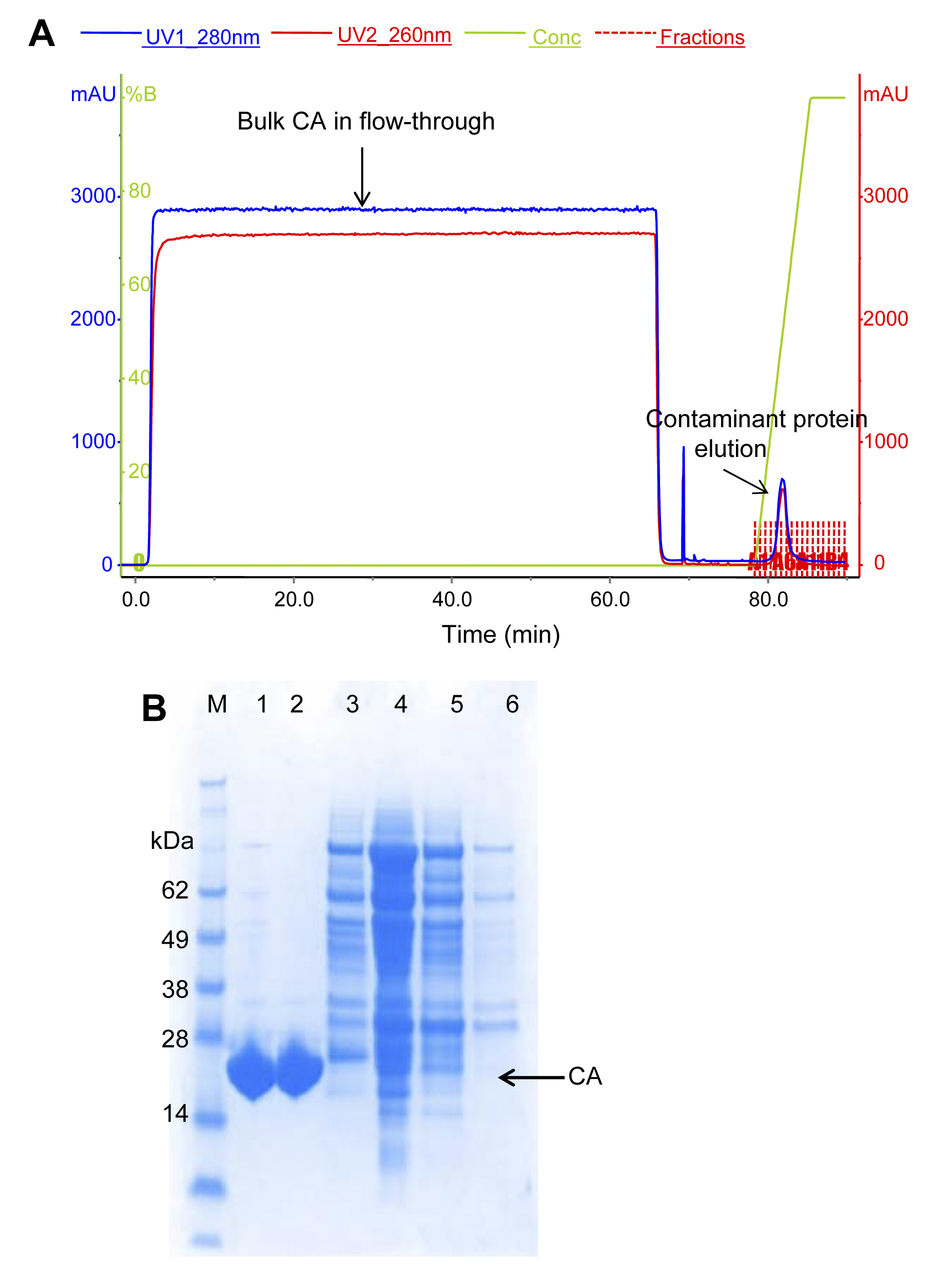

Supplement: Figure S2 — Final subtractive anion-exchange polishing step in capsid purification protocol. (A) Chromatograhic loading and elution profile of a 5 ml Q-HP Hitrap column. The column was pre-equilibrated in 50 mM sodium phosphate buffer pH 7.5 prior to loading of 400 ml of resuspended WT CA after second round of polymerization/depolymerization and dialysis. CA protein flowed through the column and was pooled. (B) SDS-PAGE analysis of protein samples collected during the anion exchange step. Lane M: SeeBluePlus 2 marker; Lane 1, Q Load; Lane 2, Q flow-through; Lanes 3–6: proteins which were captured on the column and eluted later with an increasing salt gradient. (TIF) [file pone.0058035.s002.tif]

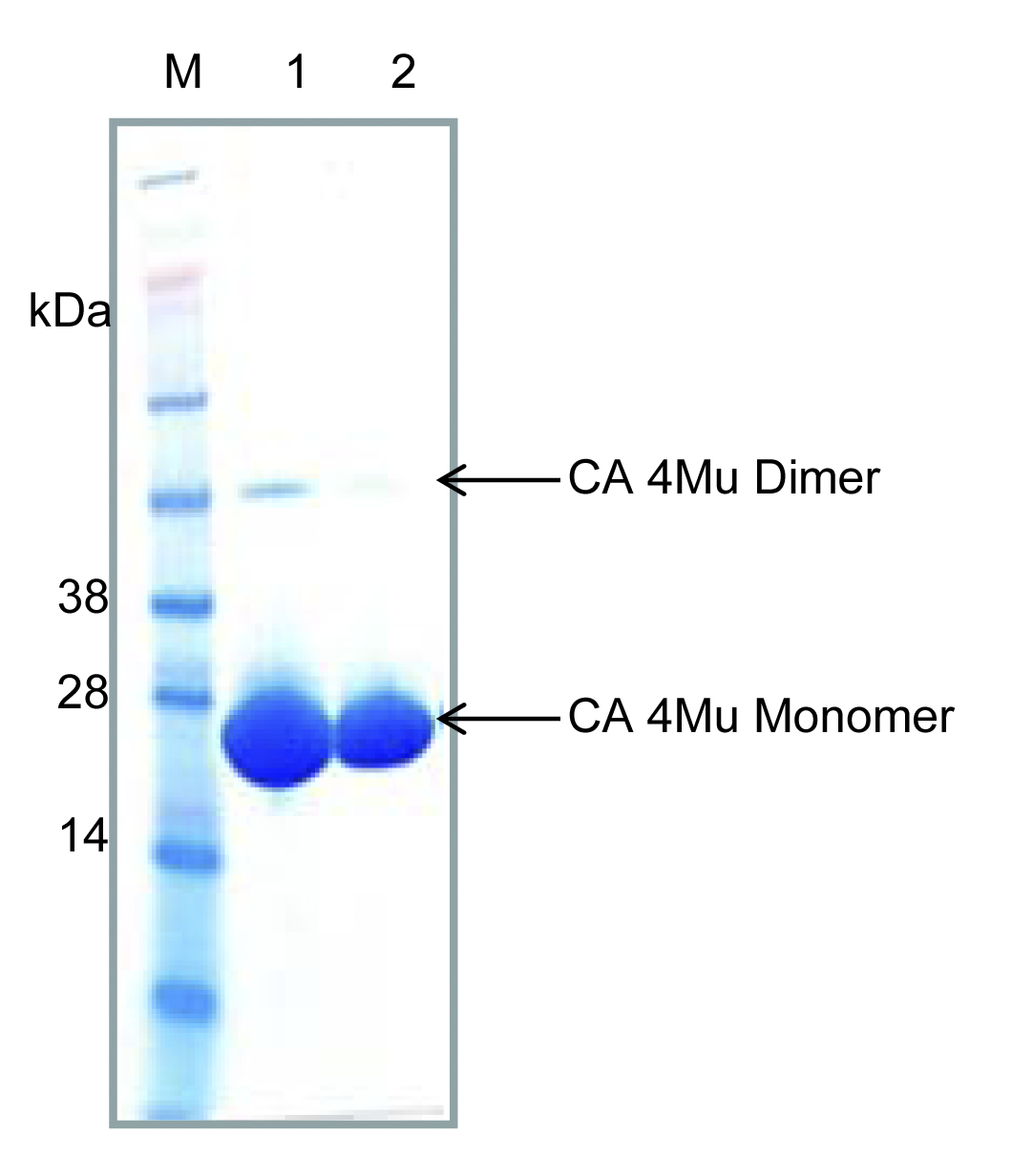

Supplement: Figure S3 — SDS-PAGE analysis of the final pool of CA 4Mu monomer ([A14C,E45C,W184A,M185A]CA). Lane M, SeeBluePlus2 Marker; Lane 1, Q column Load; Lane 2, Q-Flow-through. (TIF) [file pone.0058035.s003.tif]

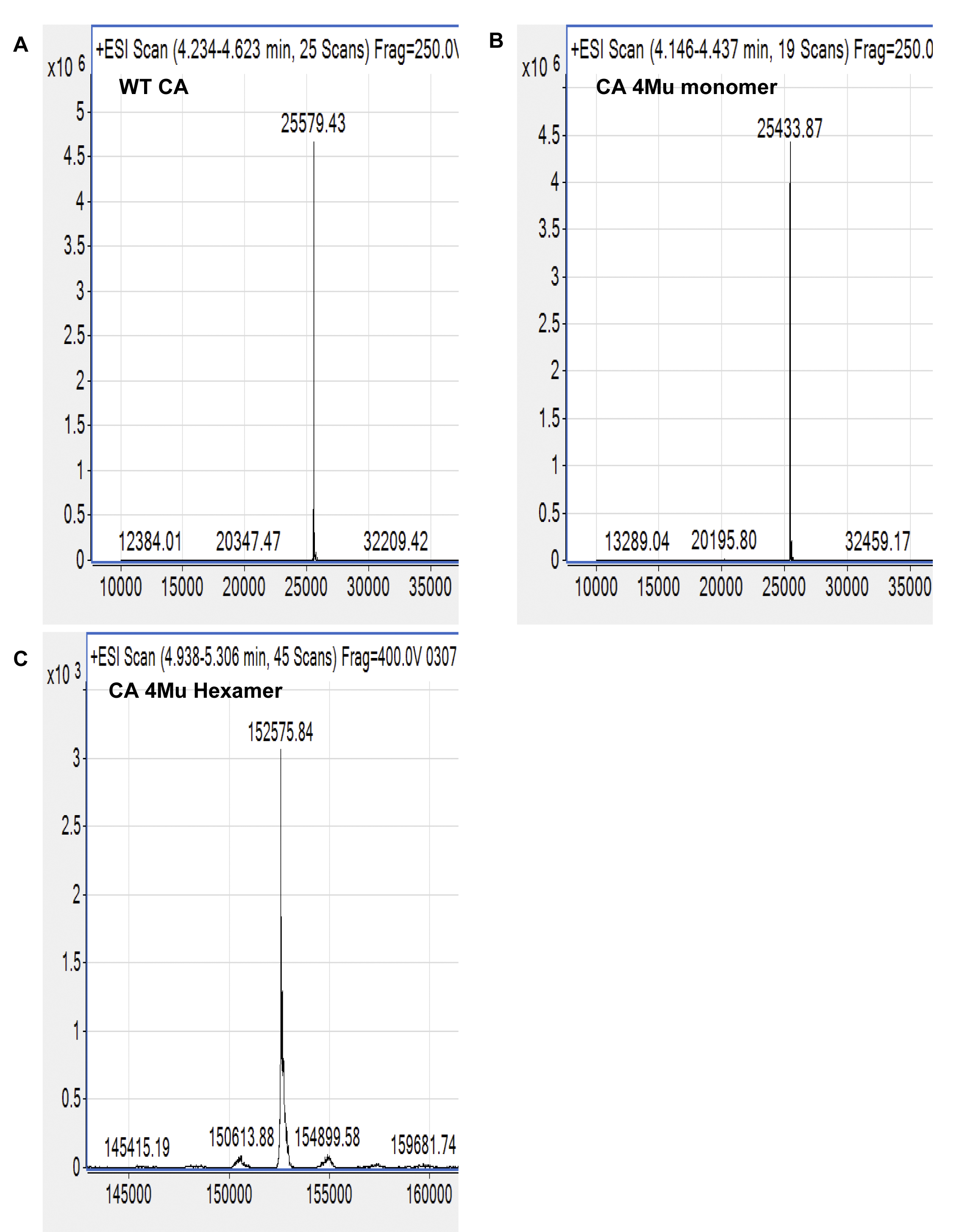

Supplement: Figure S4 — Mass spectrometry analysis of purified capsid proteins. All proteins were analyzed by ESI-ToF. Molar mass determined by mass spectrometry analysis was in excellent agreement with the mass calculated based on amino acid sequence of each protein. (A) WT CA has a calculated molecular weight of 25,579.67 Da. (B) CA 4Mu monomer has a calculated molecular weight of 25,433.55 Da. (C) CA 4Mu Hexamer has a calculated molecular weight of 152,589.3 Da, confirming the formation of 6 disulfide bond. (TIF) [file pone.0058035.s004.tif]
